# Combinatorial Therapeutic Effect of Inhibitors of Aldehyde Dehydrogenase and Mitochondrial Complex I, and the Chemotherapeutic Drug, Temozolomide against Glioblastoma Tumorspheres

**DOI:** 10.3390/molecules26020282

**Published:** 2021-01-08

**Authors:** Hun Ho Park, Junseong Park, Hye Joung Cho, Jin-Kyoung Shim, Ju Hyung Moon, Eui Hyun Kim, Jong Hee Chang, Soo Youl Kim, Seok-Gu Kang

**Affiliations:** 1 Department of Neurosurgery, Gangnam Severance Hospital, Yonsei University College of Medicine, Seoul 06273, Korea; nshhp@yuhs.ac; 2 Department of Neurosurgery, Brain Tumor Center, Severance Hospital, Yonsei University College of Medicine, Seoul 03722, Korea; j.p@catholic.ac.kr (J.P.); hjcho3633@yuhs.ac (H.J.C.); nanjk2@yuhs.ac (J.-K.S.); MJJR80@yuhs.ac (J.H.M.); euihyunkim@yuhs.ac (E.H.K.); CHANGJH@yuhs.ac (J.H.C.); 3 Precision Medicine Research Center, College of Medicine, The Catholic University of Korea, Seoul 06591, Korea; 4 Division of Cancer Biology, Research Institute, National Cancer Center, Goyang 10408, Korea; kimsooyoul@gmail.com; 5 Department of Medical Science, Yonsei University Graduate School, Seoul 03722, Korea

**Keywords:** aldehyde dehydrogenase, bioenergenetics, glioblastoma, oxidative phosphorylation, temozolomide, tumorsphere

## Abstract

Resident cancer cells with stem cell-like features induce drug tolerance, facilitating survival of glioblastoma (GBM). We previously showed that strategies targeting tumor bioenergetics present a novel emerging avenue for treatment of GBM. The objective of this study was to enhance the therapeutic effects of dual inhibition of tumor bioenergetics by combination of gossypol, an aldehyde dehydrogenase inhibitor, and phenformin, a biguanide compound that depletes oxidative phosphorylation, with the chemotherapeutic drug, temozolomide (TMZ), to block proliferation, stemness, and invasiveness of GBM tumorspheres (TSs). Combination therapy with gossypol, phenformin, and TMZ induced a significant reduction in ATP levels, cell viability, stemness, and invasiveness compared to TMZ monotherapy and dual therapy with gossypol and phenformin. Analysis of differentially expressed genes revealed up-regulation of genes involved in programmed cell death, autophagy, and protein metabolism and down-regulation of those associated with cell metabolism, cycle, and adhesion. Combination of TMZ with dual inhibitors of tumor bioenergetics may, therefore, present an effective strategy against GBM by enhancing therapeutic effects through multiple mechanisms of action.

## 1. Introduction

Despite improved standards of care, the survival rate of patients with glioblastoma (GBM) remains poor [1,2]. Surgery is the most effective treatment for complete resection, but unlike tumors of other organs, it is impossible to remove whole brain with tumors. Resident cancer cells with stem cell-like features and heterogeneity induce therapeutic tolerance and relapse, facilitating glioblastoma (GBM) survival and proliferation characterized by GBM tumorspheres (TSs) [3,4,5,6,7]. In view of the limitations of surgery alone, temozolomide (TMZ), an alkylating agent, in conjunction with postoperative radiation is employed as the standard of care for GBM. However, TMZ is insufficient for complete elimination of resistant GBM TSs. For these reasons, targeting the universal features of cancer cells is an emerging therapeutic strategy to overcome resistance to conventional cancer therapy and tumor recurrence [5,6,7,8]. Modulation of cancer cell metabolism through depletion of glucose and oxidative phosphorylation, the main source of tumor energy, is one such novel approach [9,10]. In addition, combining several therapeutic agents to inhibit multiple energy pathways may present a means to induce synergistic activity against resistant cancer cells [11,12]. Previous studies by our group showed that dual inhibition of glycolysis and oxidative phosphorylation could synergistically suppress GBM TSs [7,13,14,15]. Metformin and phenformin are biguanides reported to induce energetic stress and glucose depletion by inhibiting mitochondrial complex I and oxidative phosphorylation [7,13,14,15,16,17]. While phenformin has shown to display greater anticancer activity and tissue availability than metformin [17,18], its sole use has yielded disappointing results to date [19]. Gossypol is a polyphenolic compound extracted from cottonseed known to exert anticancer effects by inhibiting aldehyde dehydrogenase (ALDH) and oxidative phosphorylation [20,21]. Similar to phenformin, gossypol alone appears ineffective as a therapeutic agent for cancer [22,23,24].

In the present study, we aimed to overcome the weaknesses and enhance the therapeutic effects of proven agents targeting tumor bioenergetics by combination with conventional chemotherapeutic agent. The biological effects of combination drug administration with gossypol, phenformin, and TMZ compared to TMZ alone as well as gossypol and phenformin dual therapy on GBM TSs were evaluated.

## 2. Results

### 2.1. Optimization of Gossypol, Phenformin, and TMZ Concentrations

Previously, we showed that gossypol (10 μM) and phenformin (10 μM) exert dual inhibitory biological effects without affecting cell viability [15]. The sublethal concentrations of gossypol and phenformin as well as TMZ, alone and combined, were re-established using the WST assay for sphere-cultured U87 and GBM TS (TS13-64) and according to our previous findings [13]. TMZ monotherapy (250 μM), and dual therapy with gossypol (10 μM) and phenformin (10 μM) exerted minimal effects on cell viability relative to control (>50%). Combined treatment with gossypol (10 μM), phenformin (10 μM), and TMZ (250 μM) exerted significant synergistic effects on cell viability of sphere-cultured U87 and GBM TS (TS13-64).

### 2.2. Combination Therapy Inhibits Cell Proliferation and Energy Metabolism

**Figure 1:**
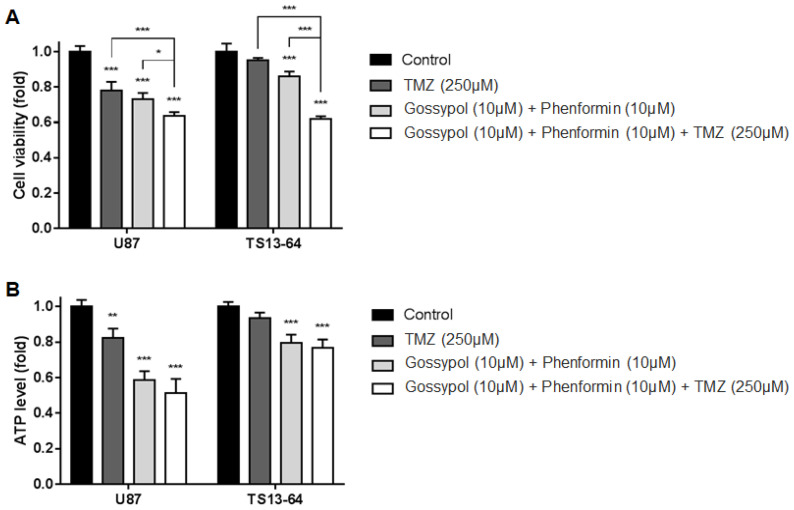
Biological effects on glioblastoma (GBM) tumorspheres (TSs) after combination drug administration of gossypol, phenformin, and temozolomide (TMZ) compared to control, TMZ monotherapy, and gossypol/phenformin dual therapy. (**A**) Cell viability and (**B**) ATP levels of U87 (*n* = 4) and TS13-64 (*n* = 4) were measured 72 h after combination drug therapy (mean ± SD; asterisks over each bar represent statistically significant differences compared to control; * *p* < 0.05, ** *p* < 0.01, *** *p* < 0.001).

TMZ monotherapy and dual therapy with gossypol and phenformin induced significant decrease in the proliferation of sphere-cultured U87 and GBM TS (TS13-64) compared to the control group. Antiproliferative effects were significantly enhanced with the combination of gossypol, phenformin, and TMZ (Figure 1A). Marked decreases in ATP levels with each agent alone and in combination, led to subsequent changes in cell viability (Figure 1B). This finding confirms that the individual agents not only exert antiproliferative effects through inhibition of cellular energy metabolism, but also that efficacy is enhanced with the combination therapy. We observed no significant differences in cell viability between TMZ monotherapy and gossypol/phenformin dual therapy. However, combination of both therapies induced significantly greater antiproliferative effects than each agent alone. Clearly, metabolic perturbations and energy stress at the cellular level need to be addressed to improve the standard of care for GBM [7,13,14,15].

### 2.3. Combination Therapy Suppresses Stemness

**Figure 2:**
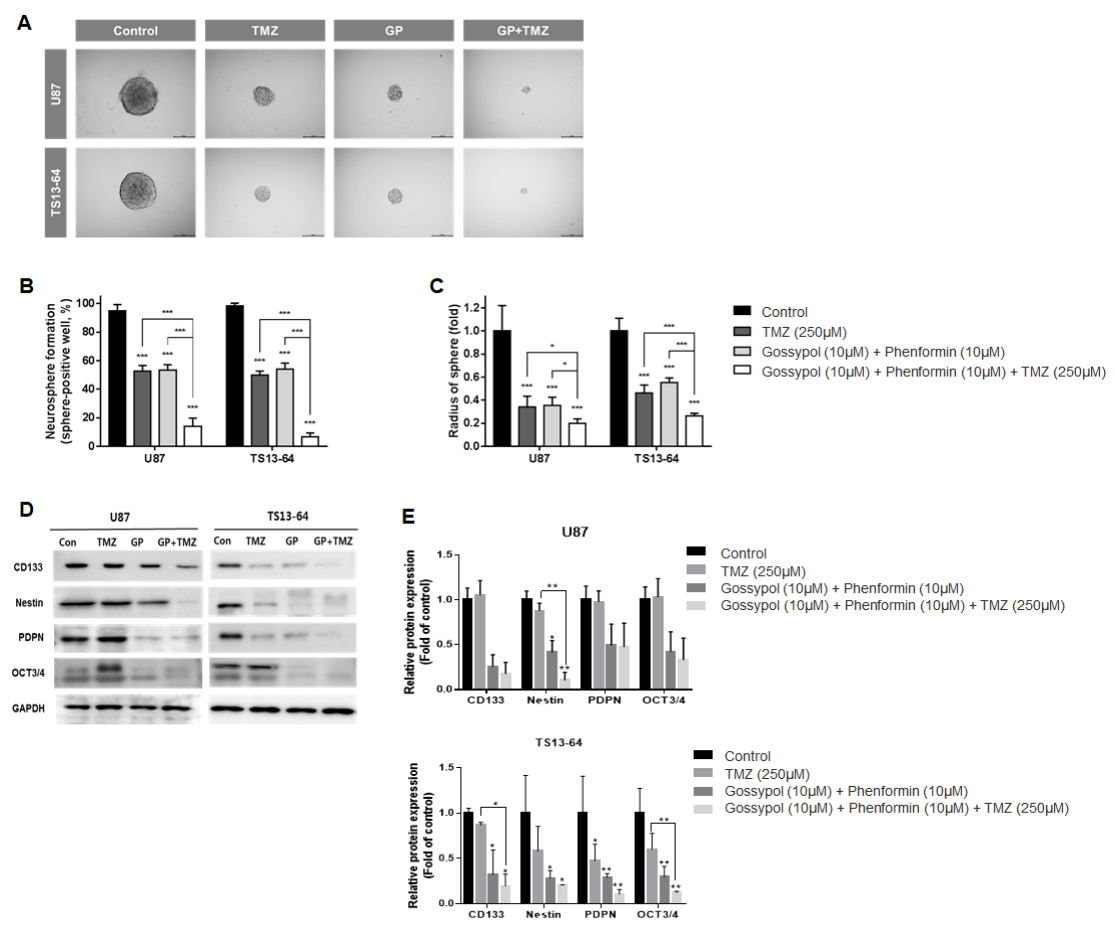
Stemness evaluation of GBM TSs after combination drug administration of gossypol, phenformin and TMZ compared to control, TMZ monotherapy, and gossypol/phenformin dual therapy. Stemness of U87 (*n* = 17) and TS13-64 (*n* = 27) was measured 3 weeks after combination drug therapy with the aid of neurosphere formation assays. (**A**) Stemness was captured using ToupView software (Toup Tek Photonics) and (**B**) quantified as a percentage of sphere-positive wells and (**C**) sphere radius (scale bar = 50 µm). (**D**) Level of protein related to stemness (CD 133, Nestin, PDPN, and Oct 3/4) were measured via western blot analysis. (**E**) Protein band intensities were quantified via densitometry. GAPDH was used as a loading control (mean ± SD; asterisks over each bar represent significant differences compared to control; * *p* < 0.05, ** *p* < 0.01, *** *p* < 0.001).

Neurosphere formation assays were used to evaluate the effects of treatment on stemness of sphere-cultured U87 and GBM TS (TS13-64) in relation to changes in the gene expression profile. TMZ monotherapy, and gossypol/phenformin dual therapy exerted equivalent stemness inhibition effects, as demonstrated by the reduced proportion of sphere-positive wells (Figure 2A). Combined treatment of gossypol and phenformin with TMZ led to remarkable enhancement of anti-stemness effects by almost completely inhibiting neurosphere formation compared to each treatment alone (Figure 2B,C). Interestingly, expression of stemness-related markers, including CD133, nestin, PDPN, and Oct3/4 was considerably reduced by gossypol, phenformin, and TMZ, both alone and combined, on western blots (Figure 2D,E). These results demonstrate that TMZ monotherapy and gossypol/phenformin dual therapy efficiently suppress stemness on their own and combining the two treatments enhances the therapeutic efficacy.

### 2.4. Combination Therapy Suppresses Invasiveness

**Figure 3:**
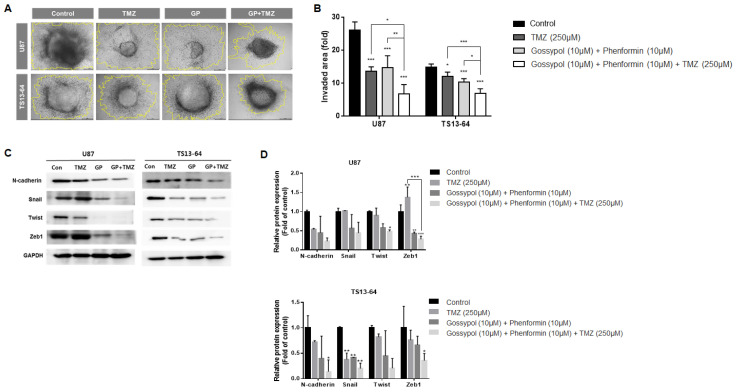
Invasiveness evaluation of GBM TSs after combination drug administration of gossypol, phenformin, and TMZ compared to control, TMZ monotherapy, and gossypol/phenformin dual therapy. Invasiveness of U87 (*n* = 5) and TS13-64 (*n* = 5) was measured 72 h after combination drug administration using 3D invasion assays. (**A**,**B**) Invasiveness was captured using ToupView software (Toup Tek Photonics) and quantified by measuring the area occupied by invading cells (outlined in yellow, scale bar = 50 µm). (**C**) Expression levels of protein related to mesenchymal transition and invasiveness (N-cadherin, Snail, Twist, and Zeb1) were measured via western blot analysis. (**D**) Protein band intensities were quantified via densitometry. GADPH was used as a loading control (mean ± SD; asterisks over each bar represent significant differences compared to control; * *p* < 0.05, ** *p* < 0.01, *** *p* < 0.001).

The invasive property of GBM TSs was evaluated using the collagen-based 3D invasion assays and quantified by assessing the area of radial migration of implanted GBM TSs into the collagen matrix. Both gossypol/phenformin and TMZ, alone and in combination induced marked suppression of invasiveness of sphere-cultured U87 and GBM TS (TS13-64) (Figure 3A). Quantitative evaluation revealed that the anti-invasive effect of gossypol and phenformin combined with TMZ was more significant than that of each therapy alone (Figure 3B). Western blot analysis of mesenchymal transition- and invasion-related markers including N-cadherin, Snail, Twist, and Zeb1, revealed substantial decrease following treatment with gossypol/phenformin and TMZ, alone or combined (Figure 3C,D). Consistently, the efficacy of TMZ monotherapy and gossypol/phenformin dual therapy could be enhanced by combining the two therapies together.

### 2.5. Transcription Profiles Following Combination Therapy

**Figure 4:**
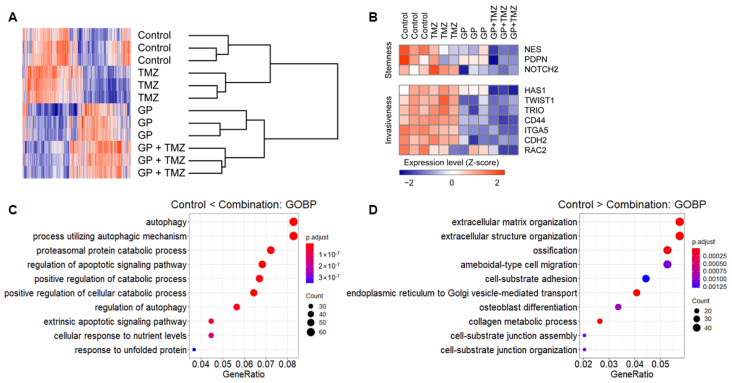
U87 cells were treated with gossypol, phenformin, and TMZ for 72 h, and gene expression profile was obtained using microarray. (**A**) For genes with average expression levels of top 30%, average linkage hierarchical clustering was performed with Euclidean distance as a distance metric, and expression levels were depicted as a heat map using GENE-E software. (**B**) Expression levels of stemness- and invasiveness-associated genes were displayed as a heat map. (**C**,**D**) Among 1799 DEGs between control and combination groups, expression levels of 837 genes, which were up-regulated in the combination group (**C**) and the expression levels of 962 genes, which were down-regulated in the combination group (**D**) were functionally annotated using GO terms.

Microarrays were used to evaluate changes in gene expression profiles after treatment with gossypol, phenformin, and TMZ. Hierarchical clustering showed strong intragroup clustering and distinct expression patterns compared with controls (Figure 4A). Notably, stemness- and invasiveness-related genes were remarkably down-regulated by gossypol and phenformin treatment, and these effects were further enhanced by TMZ combination (Figure 4B). Functional annotation of differentially expressed genes (DEGs) using GO database revealed distinct enriched gene sets. Genes up-regulated in the combination group were related to programmed cell death, autophagy, and protein catabolism, whereas down-regulated genes were associated with cell cycle and migration, which were consistent with the previous findings (Figure 4C,D). These results suggest feasible action mechanisms of the combinatorial therapeutic regimen.

## 3. Discussion

The intrinsic tendency of GBM to infiltrate normal brain tissue renders complete surgical resection of tumor an unattainable goal [25]. Several adjuvant strategies have been proposed to overcome these limitations, but none have proved successful so far [26,27,28]. Thus, adjuvant therapy targeting resident cancer cells is crucial for reversing poor survival rates. A significant subpopulation of GBM cancer cells that can survive conventional chemotherapy is proposed to possess stem cell-like features and heterogeneity [3,4,5,6,7,8]. Therapeutic tolerance and relapse of surviving cancer cells is fueled by glycolysis and oxidative phosphorylation, known to serve as major suppliers of ATP for cancer cells [29,30,31]. In view of these findings, the concept of modulating cancer metabolism by removing the energy source of tumor cells is an emerging therapeutic strategy [9,10]. Combined treatment with several therapeutic agents could induce synergistic inhibition of energy pathways [11,12]. However, successful clinical translation can only be achieved if the key molecules associated with cancer metabolism are identified for targeted therapy. Cancer cells utilize diverse nutrients, such as glucose and NADH, to fuel oxidative phosphorylation [29,30]. Therefore, induction of general metabolic stress via depletion of glucose and NADH present a reasonable approach to provide a less favorable environment for the metabolically active tumor cells [9,10].

We previously demonstrated that dual inhibition of glycolysis and oxidative phosphorylation could exert a synergistic effect with drugs against GBM TSs [7,13,14,15]. A newly designed biguanide (HL 156A) combined with TMZ [13], metformin combined with 2-deoxyglucose [14], and phenformin combined with gossypol [15], synergistically reduced ATP levels, cell viability, stemness, and invasiveness of GBM TSs. In the present study, we extended our previous research and examined the therapeutic effects of dual inhibition of glycolysis and oxidative phosphorylation with gossypol and phenformin in combination with the chemotherapeutic drug, TMZ. Biguanides such as metformin and phenformin are inhibitors of mitochondrial complex I, known to suppress cancer cell migration and proliferation [13,14,15,16,17,32]. Activated glycolytic metabolism is maintained via active glycogen synthase in GBM TSs, which could be down-regulated with inhibitors of gluconeogenesis targeting both mitochondrial and glycolytic pathways [32,33]. However, the stand alone utility of biguanides for targeting cancer metabolism is limited and their therapeutic effects against other cancers [12,19,20] and GBM TSs [13,14,15] could only be enhanced by combination with other agents. Phenformin was selected over metformin in this study owing to its superior bioavailability, potent inhibition of mitochondrial complex I, and higher CSF concentration [16,17,18]. The hydrophilic nature of metformin facilitates cellular entry specifically through organic cation transporters abundant in hepatocytes, but not elsewhere [18]. Gossypol is a naturally derived ALDH inhibitor that can suppress NADH, which fuels oxidative phosphorylation critical for metabolism of cancer cells [20,21]. Several isoforms of ALDH have been highlighted as potential drug targets, considering the elevated ALDH expression in GBM TSs [15,34]. However, similar to biguanides, gossypol on its own is ineffective against GBM TSs [15,24] and other cancers [22,23]. TMZ, a well-known conventional chemotherapeutic agent, is a crucial component of the standard of care for GBM. Unfortunately, even among TMZ-responsive patients, therapeutic tolerance and relapse can develop with consequent mortality [1,2]. According to our results, TMZ combined with gossypol and phenformin significantly and synergistically suppressed ATP levels, cell viability, stemness, and invasiveness of GBM TSs relative to treatment alone. Gossypol and phenformin induced superior ATP depletion and reduction of cell viability than TMZ while TMZ induced superior suppression of stemness and invasiveness of GBM TSs than gossypol and phenformin, albeit to a nonsignificant extent. These results support the implementation of combined therapy to overcome the weakness of stand-alone treatment and enhance the therapeutic effects of each agent in a synergistic manner. The synergistic effects of combination therapy were further supported by the functional annotation of DEGs, showing up regulation of genes associated with programmed cell death, autophagy and protein metabolism and down regulation of genes involved in cell metabolism, cycle and adhesion. The significant reduction of stemness-, mesenchymal transition- and invasion-related markers observed via western blot confirmed the above findings. The action mechanisms of gossypol/phenformin dual therapy and TMZ monotherapy are complementary, whereby the therapeutic tolerance is minimized and the synergistic efficacy maximized against GBM TSs. We anticipate to validate the results of this study through an in vivo experiment with mouse orthotopic xenograft models. The significance of the forthcoming experiments is bright considering the fact that radiotherapy could also be combined to enhance the therapeutic effects of our combinatorial therapeutic regimen.

Cancer stem cells (CSCs) are responsible for therapeutic tolerance and relapse of GBM TSs and modulation of cancer metabolism. Inhibitory effects on stemness may, therefore, serve as a promising therapeutic strategy [5,6,7]. Several reports have implicated a specific subpopulation of CSCs in invasiveness of surviving cancer cells [35,36]. However, direct evidence linking between CSCs and invasiveness is still lacking [37]. Moreover, limited information is available on GBM-specific stem cell surface markers and further research is warranted to identify potential therapeutic targets [38]. Data from the current study support our previous finding that dual inhibition of tumor bioenergetics can be effectively combined with established standard treatments. We conclude that dual inhibition of glycolysis and oxidative phosphorylation with gossypol and phenformin in combination with the chemotherapeutic drug, TMZ, presents a novel therapeutic approach against therapeutic tolerance and relapse of GBM.

## 4. Materials and Methods

### 4.1. Cell Culture and Reagents

Two TS-forming GBM lines, U87 and TS13-64 were used for study. U87 spheres were generated from the U87MG cell line under TS culture conditions. The molecular markers of U87 cell line entailed no mutation of isocitrate dehydrogenase 1 and unmethylation of O^6^-methylguanine DNA methyltransferase gene. Cells were cultured in TS complete medium composed of Dulbecco’s modified Eagle’s medium/F12 (Mediatech, Manassas, VA, USA), 1 × B27 (Invitrogen, San Diego, CA, USA), 20 ng/mL basic fibroblast growth factor, and 20 ng/mL epidermal growth factor (Sigma-Aldrich, St. Louis, MO, USA). TS-forming GBM cells (TS13-64) were established from fresh tissue specimens of a patient [13,14,15]. Detailed information of the tissue specimen entailed no mutation of isocitrate dehydrogenase 1, unmethylation of O^6^-methylguanine DNA methyltransferase gene, no loss of heterozygosity of chromosomes 1p and 19q, 20–30% positivity of p53, Ki-67 proliferation index of 40–50%, and mutation of epidermal growth factor receptor. All experiments were performed under TS culture condition. Gossypol and TMZ (MSD) were dissolved in dimethyl sulfoxide (DMSO), and phenformin (Sigma-Aldrich) in H_2_O. The treatment concentrations were as follows: 250 μM TMZ for mono treatment, 10 μM gossypol and 10 μM phenformin for dual treatment, and 10 μM gossypol, 10 μM phenformin and 250 μM TMZ for combination treatment.

### 4.2. Evaluation of ATP Level and Cell Viability

Dispersed GBM TSs were seeded in 96-well plates at a density of 104 cells/well. ATP levels were measured using a CellTiter-Glo luminescent cell viability assay kit (Promega, Fitchburg, WI, USA). A 1.0-fold ATP level was defined as the mean, normalized value in the control group. Cell viability was quantified using WST assay (Promega, Fitchburg, WI, USA).

### 4.3. Neurosphere Formation Assay

Ten dissociated, single GBM TSs were seeded in 96-well plates and cultured for three weeks with TS complete medium that was replenished every week. Images were captured and analyzed using ToupView software (version 3.7, ToupTek Photonics, Hangzhou, Zhejiang, China).

### 4.4. Invasion Assay

Each well of a 96-well plate was filled with mixed matrix composed of Matrigel, collagen type I (Corning Inc., Corning, NY, USA), and TS complete medium. U87 tumorsphere and TS13-64 cells grown as single spheroids were seeded inside the matrix prior to gelation. TS complete medium was added over the gelled matrix to prevent drying and the invasion area quantified as the occupied area at (72 h–0 h)/0 h.

### 4.5. Characterization of GBM Tumorspheres

TS formation was established from human GBM specimens as described previously [39]. GBM TSs used in the study were positive for markers of stemness, cluster of differentiation (CD) 133, and nestin (Abcam, Cambridge, UK) in immunocytochemistry. GBM TSs displayed evidence of neuroglial differentiation with expression of glial fibrillary acidic protein (GFAP) (Dako, Carpinteria, CA, USA), myelin basic protein (MBP), neuronal nuclei (NeuN), and tubulin beta 3 (TUBB3) (Chemicon, Temecula, CA, USA). GFAP and MBP could not be detected in the U87 cell line.

### 4.6. Western Blot Analysis

Cell lysates were separated via sodium dodecyl sulfate-polyacrylamide gel electrophoresis using 10% Tris-glycine gels. Protein bands were transferred to nitrocellulose membranes and probed with antibodies against CD133, Nestin (Novus Biologicals, Littleton, CO, USA), PDPN and Snail (Cell Signaling Technology, Berverly, MA, USA), N-cadherin (R&D Systems, Minneapolis, MN, USA), Zeb1 (Sigma-Aldrich), Twist, Oct3/4, and GAPDH (Santa Cruz Biotechnology, Santa Cruz, CA, USA). Detection was performed using horseradish peroxidase–conjugated IgG (Santa Cruz Biotechnology), in conjunction with Western Lightning Plus-enhanced chemiluminescence reagent (PerkinElmer, Waltham, MA, USA). Images were captured using ImageQuant LAS 4000 mini (GE Healthcare Life Sciences, Little Chalfont, UK).

### 4.7. Gene Expression Microarray Datasets and Analysis

Total RNA from GBM TSs was extracted using a Qiagen RNeasy Plus Mini kit according to the manufacturer’s protocol and loaded on the Illumina HumanHT-12 v4 Expression BeadChip column (Illumina, San Diego, CA, USA). Data were processed, transformed, and normalized with the quantile normalization method using the R/Bioconductor lumi package [40]. Using GENE-E software (Broad Institute, Cambridge, MA, USA), average linkage hierarchical clustering was performed with Pearson’s correlation as a distance metric and expression levels depicted as heat maps. The functional annotation of DEGs (one-way ANOVA with Tukey’s post hoc test; *p* < 0.001) was performed via over-representation analysis using GO Biological Process gene sets and visualized as dot plots with Bonferroni-adjusted P-value using the R/Bioconductor clusterProfiler package [41].

### 4.8. Statistical Analysis

Biological effects, stemness, and invasiveness of GBM TSs after combination drug administration (gossypol/phenformin and TMZ) compared to control, TMZ monotherapy, and gossypol/phenformin dual therapy groups were evaluated using one-way ANOVA with Tukey’s post hoc test. Average linkage hierarchical clustering was performed with Pearson’s correlation as a distance metric. Statistical significance for functional annotation of DEGs was determined using the two-sided hypergeometric test and only nodes with Bonferroni-adjusted *p* values of ≤ 0.05 displayed. All statistical analyses were performed with SPSS software version 18.0 for Windows (SAS Institute, Inc., Chicago, IL, USA). Two-tailed *p* values ≤ 0.05 were considered statistically significant.

## Data Availability

The data presented in this study are available on request from the corresponding author.

## References

[1] Stupp R., Hegi M.E., Mason W.P., ven den Bent M.J., Taphoorn M.J.B., Janzer R.C., Ludwin S.K., Allgeier A., Fisher B., Belanger K. (2009). Effects of radiotherapy with concomitant and adjuvant temozolomide versus radiotherapy alone on survival in glioblastoma in a randomized phase III study: 5-year analysis of the EORTC-NCIC trial. Lancet Oncol..

[2] Stupp R., Mason W.P., van den Bent M.J., Weller M., Fisher B., Taphoorn M.J.B., Belanger K., Brandes A.A., Marosi C., Bogdahn U. (2005). Radiotherapy plus concomitant and adjuvant temozolomide for glioblastoma. N. Engl. J. Med..

[3] Jackson M., Hassiotou F., Nowak A. (2014). Glioblastoma stem-like cells: At the root of tumor recurrence and a therapeutic target. Carcinogenesis.

[4] Auffinger B., Spencer D., Pytel P., Ahmed A.U., Lesniak M.S. (2015). The role of glioma stem cells in chemotherapy resistance and glioblastoma multiforme recurrence. Expert Rev. Neurother..

[5] Lee J.H., Lee J.E., Kahng J.Y., Kim S.H., Park J.S., Yoon S.J., Um J.Y., Kim W.K., Lee J.K., Park J. (2018). Human glioblastoma arises from subventricular zone cells with low-level driver mutations. Nature.

[6] Yoon S.-J., Park J., Jang D.-S., Kim H.J., Lee J.H., Jo E., Choi R.J., Shim J.-K., Moon J.H., Kim E.-H. (2019). Glioblastoma cellular origin and the firework pattern of cancer genesis from the subventricular zone. J. Korean Neurosurg. Soc..

[7] Lim E.-J., Kim S., Oh Y., Suh Y., Kaushik N., Lee J.-H., Lee H.-J., Kim M.-J., Park M.-J., Kim R.-K. (2020). Crosstalk between GBM cells and mesenchymal stemlike cells promotes the invasiveness of GBM through the C5a/p38/ZEB1 axis. Neuro Oncol..

[8] Kim S.-Y. (2015). Cancer metabolism: Targeting cancer universality. Arch. Pharmacal Res..

[9] Bobrovnikova-Marjon E., Hurov J.B. (2014). Targeting metabolic changes in cancer: Novel therapeutic approaches. Annu. Rev. Med..

[10] Kim S.-Y. (2015). Cancer metabolism: Strategic diversion from targeting cancer drivers to targeting cancer suppliers. Biomol. Ther..

[11] Cheng G., Zielonka J., McAllister D.L., Tsai S., Dwinell M.B., Kalyanaraman B. (2014). Profiling and targeting of cellular bioenergetics: Inhibition of pancreatic cancer cell proliferation. Br. J. Cancer.

[12] Ben Sahra I., Tanti J.-F., Bost F. (2010). The combination of metformin and 2 deoxyglucose inhibits autophagy and induces AMPK-dependent apoptosis in prostate cancer cells. Autophagy.

[13] Choi J., Lee J.-H., Koh I., Shim J.-K., Park J., Jeon J.Y., Hyun K.E., Kim S.H., Yook J.I., Kim E.H. (2016). Inhibiting stemness and invasive properties of glioblastoma tumorsphere by combined treatment with temozolomide and a newly designed biguanide (HL156A). Oncotarget.

[14] Kim E.H., Lee J.-H., Oh Y., Koh I., Shim J.-K., Park J., Choi J., Yun M., Jeon J.Y., Huh Y.M. (2016). Inhibition of glioblastoma tumorspheres by combined treatment with 2-deoxyglucose and metformin. Neuro Oncol..

[15] Park J., Shim J.-K., Kang J.H., Choi J., Chang J.H., Kim S.-Y., Kang S.-G. (2017). Regulation of bioenergetics through dual inhibition of aldehyde dehydrogenase and mitochondrial complex I suppresses glioblastoma tumorspheres. Neuro Oncol..

[16] Pollak M. (2013). Potential applications for biguanides in oncology. J. Clin. Investig..

[17] Owen M.R., Doran E., Halestrap A.P. (2000). Evidence that metformin exerts its anti-diabetic effects through inhibition of complex 1 of the mitochondrial respiratory chain. Biochem J..

[18] Pernicova I., Korbonits M. (2014). Metformin—mode of action and clinical implications for diabetes and cancer. Nat. Rev. Endocrinol..

[19] Shackelford D.B., Abt E., Gerken L., Vasquez D.S., Seki A., Leblanc M., Wei L., Fishbein M.C., Czernin J., Mischel P.S. (2013). LKB1 Inactivation Dictates Therapeutic Response of Non-Small Cell Lung Cancer to the Metabolism Drug Phenformin. Cancer Cell.

[20] Kang J.H., Lee S.-H., Lee J.-S., Nam B., Seong T.W., Son J., Jang H., Hong K.M., Lee C., Kim S.-Y. (2016). Aldehyde dehydrogenase inhibition combined with phenformin treatment reversed NSCLC through ATP depletion. Oncotarget.

[21] Koppaka V., Thompson D.C., Chen Y., Ellermann M., Nicolaou K.C., Juvonen R.O., Petersen D., Deitrich R.A., Hurley T.D., Vasiliou V. (2012). Aldehyde dehydrogenase inhibitors: A comprehensive review of the pharmacology, mechanism of action, substrate specificity, and clinical application. Pharmacol. Rev..

[22] Van Poznak C., Seidman A.D., Reidenberg M.M., Moasser M.M., Sklarin N., Van Zee K., Borgen P., Gollub M., Bacotti D., Yao T.-J. (2001). Oral Gossypol in the treatment of patients with refractory metastatic breast cancer: A phase I/II clinical trial. Breast Cancer Res. Treat..

[23] Ready N.E., Karaseva N.A., Orlov S.V., Luft A.V., Popovych O., Holmlund J.T., Wood B.A., Leopold L. (2011). Double-blind, placebo-controlled, randomized phase 2 study of the proapoptotic agent AT-101 plus docetaxel, in second-line non-small cell lung cancer. J. Thorac. Oncol..

[24] Bushunow P., Reidenberg M.M., Wasenko J., Winfield J., Lorenzo B., Lemke S., Himpler B., Corona R., Coyle T. (1999). Gossypol treatment of recurrent adult malignant gliomas. J. Neuro Oncol..

[25] Tanahashi K., Natsume A., Ohka F., Momota H., Kato A., Motomura K., Watabe N., Muraishi S., Nakahara H., Saito Y. (2014). Assessment of tumor cells in a mouse model of diffuse infiltrative glioma by raman spectroscopy. BioMed Res. Int..

[26] Thomas A.A., Brennan C.W., DeAngelis L.M., Omuro A.M. (2014). Emerging therapies for glioblastoma. JAMA Neurol..

[27] Chinot O.L., Wick W., Mason W., Henriksson R., Saran F., Nishikawa R., Carpentier A.F., Hoang-Xuan K., Kavan P., Cernea D. (2014). Bevacizumab plus radiotherapy-temozolomide for newly diagnosed glioblastoma. N. Engl. J. Med..

[28] Gilbert M.R., Dignam J.J., Armstrong T.S., Wefel J.S., Blumenthal D.T., Vogelbaum M.A., Colman H., Chakravarti A., Pugh S., Won M. (2014). A randomized trial of bevacizumab for newly diagnosed glioblastoma. N. Engl. J. Med..

[29] Schulze A., Harris A.L. (2012). How cancer metabolism is tuned for proliferation and vulnerable to disruption. Nat. Cell Biol..

[30] Zu X.L., Guppy M. (2004). Cancer metabolism: Facts, fantasy, and fiction. Biochem. Biophys. Res. Commun..

[31] Warburg O., Wind F., Negelein E. (2004). The metabolism of tumors in the body. J. Gen. Physiol..

[32] Bao B., Wang Z., Ali S., Ahmad A., Azmi A.S., Sarkar S.H., Banerjee S., Kong D., Li Y., Thakur S. (2012). Metformin inhibits cell proliferation, migration and invasion by attenuating CSC function mediated by deregulating miRNAs in pancreatic cancer cells. Cancer Prev. Res..

[33] Beckner M.E., Gobbel G.T., Abounader R., Burovic F., Agostino N.R., Laterra J., Pollack I.F. (2005). Glycolytic glioma cells with active glycogen synthase are sensitive to PTEN and inhibitors of PI3K and gluconeogenesis. Lab. Investig..

[34] Nakano I. (2015). Stem cell signature in glioblastoma: Therapeutic development for a moving target. J. Neurosurg..

[35] Korkaya H., Paulson A., Iovino F., Wicha M.S. (2008). HER2 regulates the mammary stem/progenitor cell population driving tumorigenesis and invasion. Oncogene.

[36] Wicha M.S., Liu S., Dontu G., Banks R.E., Tirukonda P., Taylor C., Hornigold N., Astuti D., Cohen D., Maher E.R. (2006). Cancer stem cells: An old idea-A paradigm shift. Cancer Res..

[37] Ortensi B., Setti M., Osti D., Pelicci G. (2013). Cancer stem cell contribution to glioblastoma invasiveness. Stem Cell Res. Ther..

[38] Jordan C.T. (2009). Cancer stem cells: Controversial or just misunderstood?. Cell Stem Cell.

[39] Kong B.H., Park N.-R., Shim J.-K., Kim B.-K., Shin H.-J., Lee J.-H., Huh Y., Lee S.-J., Kim S.H., Kim E. (2012). Isolation of glioma cancer stem cells in relation to histological grades in glioma specimens. Child. Nerv. Syst..

[40] Du P., Kibbe W.A., Lin S.M. (2008). lumi: A pipeline for processing Illumina microarray. Bioinformatics.

[41] Yu G., Wang L.-G., Han Y., He Q.-Y. (2012). clusterProfiler: An R package for comparing biological themes among gene clusters. OMICS J. Integr. Biol..

